# Mortality trends among migrant groups living in Amsterdam

**DOI:** 10.1186/s12889-015-2523-6

**Published:** 2015-11-27

**Authors:** Daan G Uitenbroek

**Affiliations:** Municipal Health Service, Nieuwe Achtergracht 100, P O Box 2200, 1000 CE Amsterdam, The Netherlands

**Keywords:** Immigration, Migrant mortality, Migrant life expectancy, Social change, Amsterdam, The Netherlands

## Abstract

**Background:**

The main aim of this paper is to see to what extent mortality patterns between migrants living in the Netherlands converge. This might be an indicator of health and health care acculturation.

**Methods:**

This is an observational study on the basis of standard mortality registration data collected between 1996 and 2007. Eight ethnic groups living in Amsterdam are examined to see if mortality converges or diverges over time. Trends in mortality are studied using Poisson regression. The life expectancy between groups is compared for three time periods.

**Results:**

The data showed that for males and females the life expectancy and death rates improved between 1996–1999 and 2004–2007. Most ethnic groups, both males and females, followed this positive trend. For most indicators the ethnic groups converged in terms of mortality. The data also shows the healthy migrant effect with those in Amsterdam from Dutch origin having a relatively high mortality and low life expectancy.

**Discussion:**

In this paper the “healthy migrant effect” can be clearly observed. An important cause is the emigration of the original and relatively affluent and healthy Dutch population to suburban areas. Mortality trends tend to converge between ethnic groups during the period 1997–2000 and 2004–2007. The data presented here shows further that trends in mortality and life expectancy which apply to all ethnic groups are much more powerful as this convergence. One wonders if bridging the mortality gap between groups is of much benefit for minority groups, or that minority groups would benefit more from an overall decrease in mortality.

**Conclusion:**

Mortality trends that apply to all groups tend to be much stronger compared with trends for individual groups. This shows that dynamics affecting all groups similarly have a considerably stronger effect on mortality outcomes in various ethnic groups compared with possible convergence.

**Convergence of mortality trends among ethnic groups living in Amsterdam**

## Background

To what extent processes of adaptation and integration lead to a convergence of health and health behaviour in societies with a variety of migrant groups is an important issue in public health. Although it might take many different forms, migrants will always adapt to or “acculturate” into the society into which they migrate to some extent. Migrating involves contact and participation in the new society while, at the same time, culture and identity will be maintained [1, 2]. If the host society is “welcoming”, the levels of suspicion between social groups, a wish to maintain a distinct identity, (un)equal access to care, are factors [3–7]. This paper concentrates on differences in mortality as an indicator of differences of changes in health experience between migrant groups. Mortality is a limited indicator that fails to fully appreciate differences in health and morbidity, health behaviour, and care use. However, differences between social groups in mortality are a sure indicator of differences in underlying health aspects, and can be a valuable starting point for further study.

A distinction by Graves [1, 8] sees acculturation in terms of an individual and psychological process and acculturation as a group and cultural process. In the first type of study, migrants are followed to see if their health changes with longer duration of stay and developing personal acculturation. These studies are difficult as duration-of-stay effects are hard to separate from ageing effects. An example of a solution of this problem is a study in which the mortality of non-migrating twins in Finland was compared with the mortality of their migrating sibling in Sweden [9]. In general, the mortality experience of the migrants remained comparable with their sibling living in Finland; some adaptation to the higher standard of living in Sweden with improving mortality regarding a number of causes took place. Bos et al., [10] studied the mortality divide between migrants with various durations of stay compared with the resident Dutch population in an age-standardized design. Only a limited relation between duration of stay and the mortality divide was found. In a controlled study it is found that with increasing length of stay the health advantage of migrants of Asian and Pacific origin decreased [11].

A second line of inquiry asks the question how migrants categorized in different groups by origin perform with regard to their collective “average” health over time. Singh and Hiatt [12] compared the mortality of U.S citizens who were born in the U.S. with U.S. citizens who were not born in the U.S. The mortality advantage of those not born in the U.S. widened further over time. This was mainly due to the influx of migrants with a mortality advantage compared with U.S. born citizens. In the US racial gaps in life expectancy decreased during the last decades [13–15]. This decrease is partly masked by an increase in the educational gap, lower educated groups improving their life expectancy less than higher educated groups [13]. The decease is caused primarily by a decrease in the age gap of dying which exists between the various group [16], and trends are very different in the different US states [17]. Garssen and van der Meulen [16] found that mortality differences between various migrant groups and the Dutch population are decreasing over time.

This paper is about changes in mortality in eight ethnic groups living in Amsterdam during the periods 1997–2000, 2001–2003 and 2004–2007. The mortality and life expectancy of these groups is compared with changes in mortality in Amsterdam in general and changes in mortality in the Netherlands. The analysis is disaggregated into the age groups 15–45 and 45–65. The age group 15–45 is selected because causes linked to high social activity, such as infectious diseases and trauma mortality such as accidents, suicides and homicides, are important. The age group 45–65 is selected because chronic diseases related to inherited characteristics, lifestyle, and prevention, are very important. Studies of differences in mortality show that in the Netherlands trauma mortality, particularly homicide, is much more common in immigrant groups, while cancers are less common overall [10, 16]. For the age group 65+ only the life table is presented.

Lastly, a thorough discussion of Dutch immigration patterns as a background to reading this paper is found elsewhere [17].

## Methods

### Data set

The data consists of information collected by the civil registry in Amsterdam concerning the population size and number of deaths for the city summarized over the periods 1997–2000, 2001–2003 and 2004–2007 and was provided in table form by the Office for Research and Statistics of the city of Amsterdam. The number of deaths and population data relate exclusively to people legally residing in Amsterdam. In the context of combating tax and benefit fraud there is a major effort to make this data complete. Registrations from the city of Amsterdam, the tax authorities, housing associations and utility companies are included in this effort. Returned, non-answered and inappropriately answered mail is followed-up by house visits, visits to family members and neighbours. The municipal personal records database, on which the mortality and population of this study is based, is therefore generally considered to be complete and up to date. However, there will be differences between origin groups in terms of this completeness, which may influence the results of this study.

Origin is determined in the following way: each citizen is classified as a member of a certain nationality group, being considered of foreign origin if the individual himself or herself or one or both of the parents are born abroad. In case of mixed parentage precedence is given to the parent from Southern or Eastern Europe or a non-industrialised nation for classification. In the case of parentage of mixed nationality with both parents from Southern or Eastern Europe or a non-industrialised country, the mother's nationality is used for classification. The classification is in accordance with national guidelines [18] and results in eight ethnicity groups: Dutch; Industrialized; Non Industrialized; Southern European; Moroccan; Turkish; Antillean; Surinam. Number of person years and deaths for each of the groups for each of periods used in the analysis are shown in table 1. This way the most important migrant groups living in the Netherlands are separately classified while the smaller groups are clustered in three groups: (non) industrialized and Southern European.

**Table 1:** Person time and (number death), Amsterdam 1996–2007

	*Males*			*Females*		
	2004-2007	2000-2003	1996-1999	2004-2007	2000-2003	1996-1999
Dutch	730412 (8074)	748899,5 (9439)	776147 (10230)	774528,5 (9884)	804699,5 (11614)	849391,5 (12245)
Industrialized	140511,5 (1015)	137629,5 (1098)	131266 (1125)	147533 (1268)	145772,5 (1465)	142587 (1485)
Non Industrialized	183294 (426)	165688,5 (371)	137854,5 (363)	178558 (294)	152573 (325)	122944 (275)
Southern European	37366,5 (158)	36673,5 (147)	35261 (133)	34573 (76)	33067 (61)	30840,5 (52)
Moroccan	136866,5 (312)	125415,5 (245)	110856 (188)	125838 (126)	111062 (114)	93501,5 (83)
Turkish	80300,5 (211)	75609 (175)	68951,5 (161)	73776,5 (93)	68092 (87)	59259 (61)
Antillean	23793,5 (72)	23937 (92)	21648 (59)	23848,5 (53)	24412,5 (60)	22017,5 (42)
Surinamese	131731,5 (577)	135389,5 (588)	133796,5 (499)	149177 (530)	152018,5 (546)	148701,5 (457)
City of Amsterdam	1464276 (10845)	1449242 (12155)	1415780,5 (12758)	1507832,5 (12324)	1491697 (14272)	1469242,5 (14700)
Netherlands	32310893,5 (262812)	31843408,5 (275102)	30984451 (273332)	33018550 (278537)	32502514,5 (290093)	31675992,5 (277981)

### Statistical analysis

The analysis is done in “R” (http://www.r-project.org/) using Poisson regression analysis with the death numbers as a variable outcome and the factor “ethnicity” and the covariate years as independent variables. The population size is the offset variable. The analysis is done separately for males and females. Over- or under dispersion, wrongly estimating the variance and thus over- or underestimating the statistical significance of differences is a problem in Poisson regression analysis. Using a negative binomial regression, were the variance is estimated separate from the mean, is a possible solution. However, the parameters of the Poisson regression are reported in this paper because they are easier to interpret. Whenever there are results near the critical value (z ~ 2) the Poisson is compared with the negative binomial model to check for possible dispersion problems. This is discussed in the result section. The life table analysis is according to Chiang’s method [19]; the spread sheet used is available on the SISA website http://www.quantitativeskills.com/downloads/#Lifetab. Standard deviations for the differences between the groups in the measurements are presented; these were calculated using Ms-Excel. Increasing standard deviations point to the groups showing more differences. The F-test is used to test for the significance of the difference between two standard deviations.

### Privacy and ethics

The data concerns aggregated and tabulated anonymised publicly available data and there are no privacy concerns. Ethical approval is not required. There is no external funding and there are no competing interests.

## Results

Table 2 shows the life expectancy at birth for the total population of Amsterdam and the Netherlands, eight ethnic groups, for three different time periods. The life expectancy gives a good overview of differences in mortality over the complete life cycle. The table shows that among both males and females, and both in Amsterdam and the Netherlands, the life expectancy increases over time, with a particularly strong increase in males and in the city of Amsterdam. The result of these patterns is that the mortality disadvantage for males and for the city of Amsterdam compared with females and the Netherlands is decreasing.

**Table 2:** Life expectancy at birth, Amsterdam 1996–2007 (95 % confidence interval)

	*Males*			*Females*		
	2004-2007	2000-2003	1996-1999	2004-2007	2000-2003	1996-1999
Dutch	76.4 (76.1-76.7)	74.5 (74.2-74.9)	73.6 (73.3-73.9)	80.7 (80.4-80.9)	79.5 (79.2-79.8)	79.1 (78.8-79.4)
Industrialized	77.2 (76.5-77.9)	75.5 (74.9-76.1)	73.6 (72.8-74.4)	81.2 (80.5-81.8)	79.4 (78.7-80.1)	79.6 (78.8-80.3)
Non Industrialized	79.3 (78.2-80.3)	77.4 (76.3-78.5)	75.3 (74.0-76.6)	85.2 (84.1-86.3)	81.5 (80.4-82.5)	81.0 (79.8-82.2)
Southern European	77.9 (76.6-79.3)	77.5 (75.8-79.2)	77.2 (75.1-79.2)	85.3 (83.5-87.1)	84.5 (82.9-86.0)	86.6 (84.2-88.9)
Moroccan	86.0 (84.4-87.5)	79.6 (78.2-80.9)	80.1 (78.4-81.9)	86.2 (84.7-87.7)	84.6 (82.0-87.2)	101.7 (96.6-106.7)
Turkish	75.7 (74.4-77.0)	76.2 (74.1-78.4)	72.8 (70.9-74.7)	82.7 (81.3-84.0)	82.4 (80.4-84.4)	100.9 (95.7-106.0)
Antillean	75.5 (73.0-78.0)	73.3 (70.1-76.5)	77.0 (73.4-80.6)	84.2 (81.9-86.6)	82.6 (79.5-85.7)	81.4 (78.7-84.1)
Surinamese	76.2 (75.3-77.1)	74.7 (73.7-75.6)	74.4 (73.3-75.5)	82.2 (81.4-83.1)	80.0 (79.1-80.9)	80.5 (79.6-81.5)
Standard dev.	3.46	2.02	2.45	2.05	2.09	9.51
City of Amsterdam	76.6 (76.3-76.8)	74.7 (74.5-75.0)	73.7 (73.5-74.0)	81.0 (80.8-81.2)	79.6 (79.4-79.8)	79.3 (79.1-79.5)
Netherlands	77.8 (77.8-77.8)	76.1 (76.1-76.2)	75.3 (75.2-75.3)	82.4 (82.3-82.4)	81.0 (81.0-81.1)	80.8 (80.8-80.9)

Table 2 further shows that for both males and females in all three periods citizens of Dutch descent have a slightly lower life expectancy compared with the average for Amsterdam. The confidence intervals show that these differences are not statistically significant. For citizens of Moroccan, Southern European and Industrialized descent the life expectancy is higher compared with Amsterdam in all three periods, for females this is further the case for citizens of Turkish, Antillean and Surinamese origin. Change patterns over time are difficult to judge, as they fluctuate quite strongly. Small numbers of deaths in some periods in some ethnic groups are a factor in this. Generally speaking, citizens of Dutch descent have a relatively low life expectancy, their life expectancy is improving but at an average rate. Also among migrants from industrialized countries and Surinam the life expectancy is consistently increasing. In the table the standard deviation of the life expectancies for the eight groups is presented. An increase in the standard deviation indicates that the groups become more different over time with regard to life expectancy, a decrease indicates less difference. The standard deviation for males increased from 2.5 in 1996–1999 to 3.5 in 2004–2007 (F = 1.96; p = 0.18), while for females it decreased from 9.5 to 2.1 (F = 20.5; p < 0.01).

**Fig. 1:**
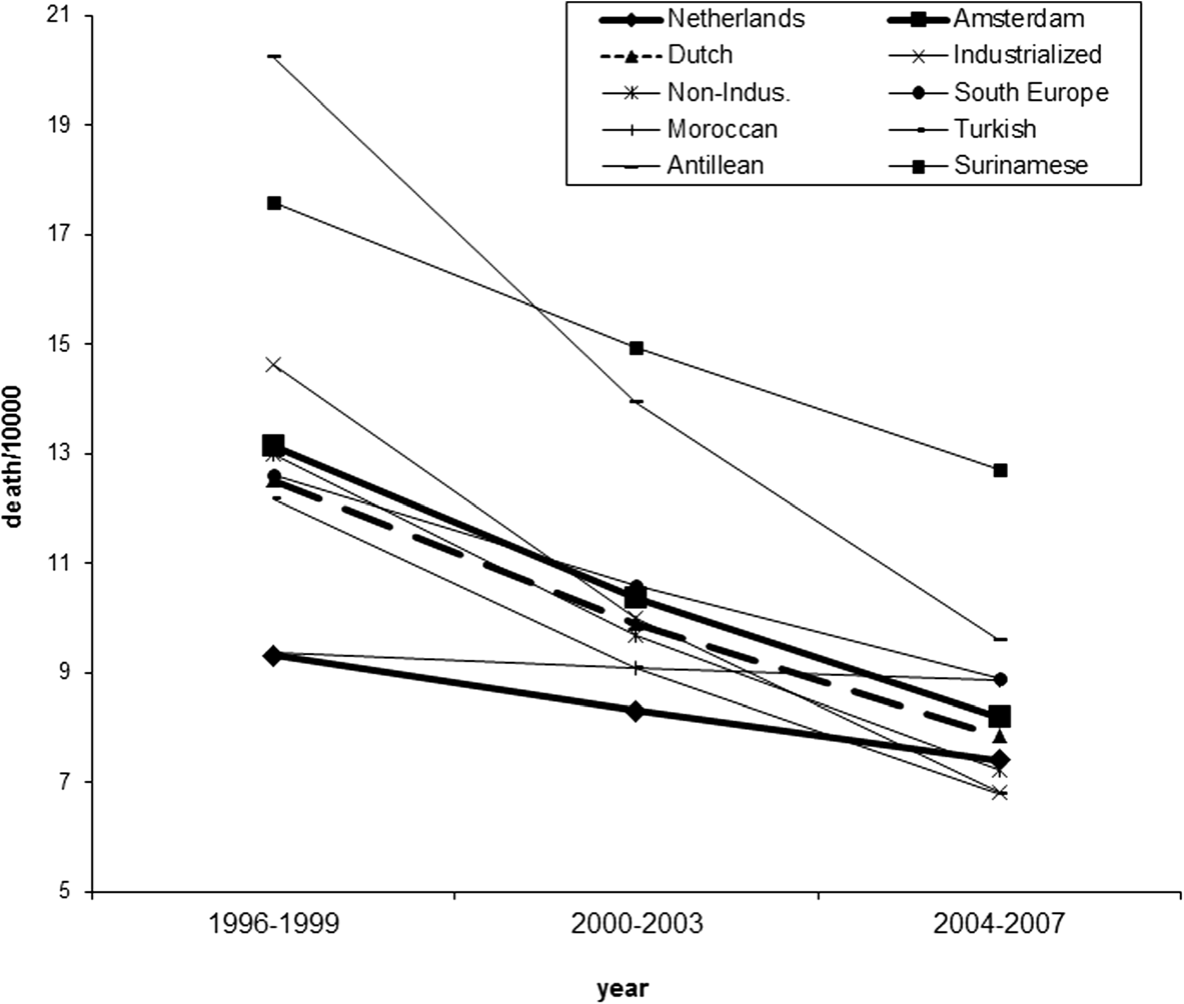
Chances of dying per 10,000 population for males aged between 15 and 45

Figure 1 shows the estimated trend lines for the chances of dying per 10,000 population for males aged between of 15 and 45. The bottom bold line shows the trend for males in the Netherlands, the top bold line the trend for males in Amsterdam, the dashed bold line the trend for citizens of Amsterdam of Dutch origin. The thin lines are trends for the other seven ethnic groups. The key between numbers and groups can be found in the second column of Table 2. The figure shows that both the number of deaths in these age groups for Amsterdam and for the Netherlands in general decrease very considerably, this downward trend is significant in both the Netherlands (z = 17.3; p < 0.01) and in Amsterdam (z = 9,2; p < 0.01) whereby mortality decreases significantly stronger in Amsterdam (z = 4.6; p < 0.01). All ethnic groups have this downward trend. As shown in the figure, extreme groups tend to converge to the mean over time, this is particularly the case for Moroccans who, in 1996–1999, had a relatively favourable mortality and who are improving relatively little, and males from Surinam, who had a relatively unfavourable mortality, and who improve their mortality a lot. Comparing the trend for all ethnic groups with the average for Amsterdam, only the trend for males from Morocco was statistically significantly different (z = 2.1; p = 0.03). There was no difference between the Poisson and the negative binomial model (Chi2 = 0.68; p = 0.41). Lastly, the standard deviation of the death rates over the eight ethic groups (not presented in the figure or a table) decreased from 3.46 in 1996–1999 to 1.96 in 2004–2007 (F = 3.12; p = 0.06), showing a (borderline significant) convergence of mortality patterns over time.

**Fig. 2:**
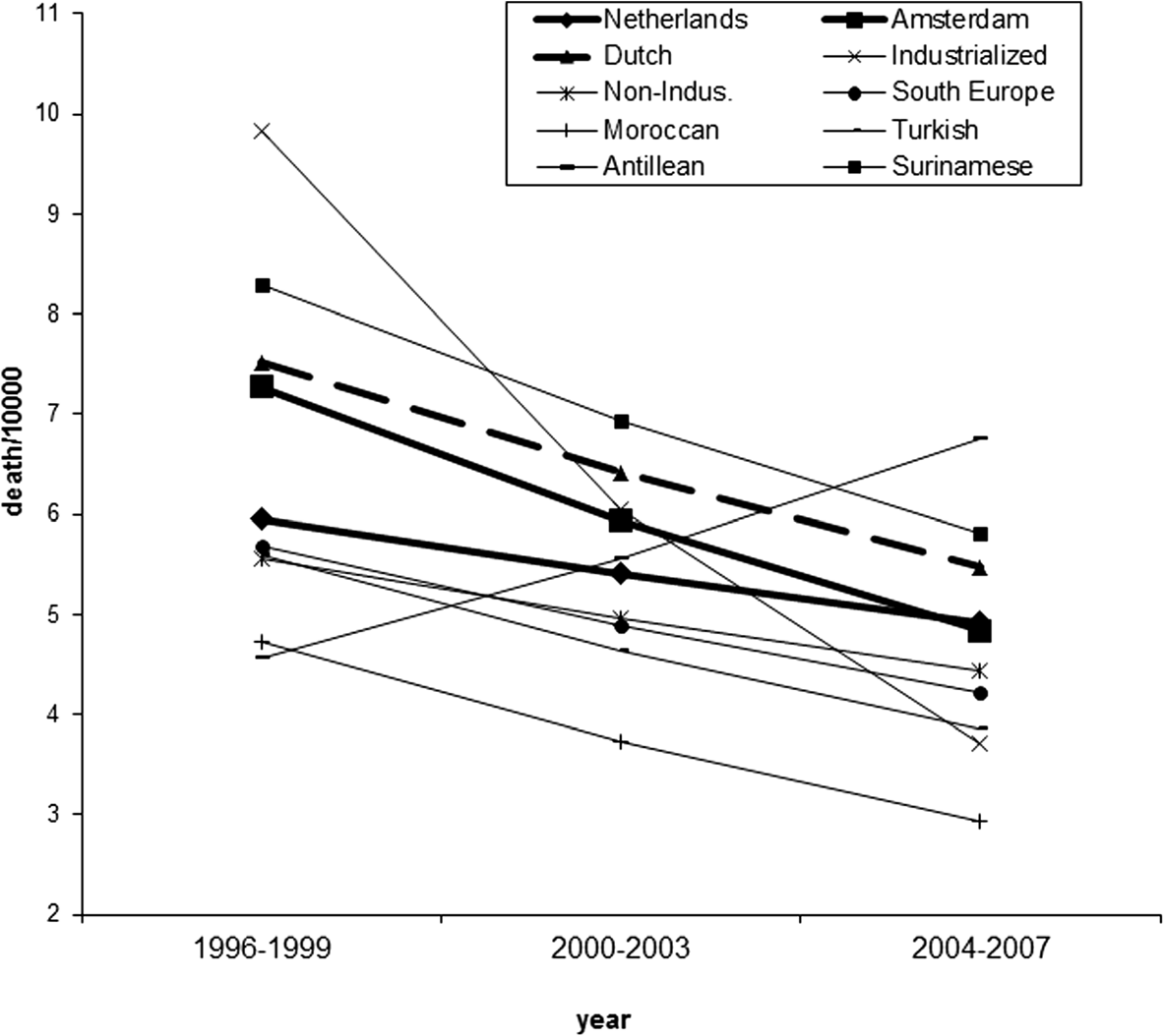
Chances of dying per 10,000 females aged between 15 and 45

Figure 2 shows the same pattern for females. Again mortality dropped statistically significantly both in the Netherlands (z = 11.5; p < 0.01) and in Amsterdam (z = 6.0; p < 0.01), significantly stronger so in Amsterdam (z = 3.1; p < 0.01). With the exception of females from the Antilles all ethnic groups follow this trend. Yet again, the most extreme groups change most. Among females from the industrialized countries, who have a very high mortality in 1996–1999, mortality went down sharply, while among females from the Antilles mortality went up sharply. Only the trend of the industrialized countries was significantly different compared with the average trend for Amsterdam (z = 2.5; p < 0.01). The negative binomial regression produced similar results compared with the Poisson regression (Chi2 < 0.01; p = 0.98). The standard deviation for the death rates decreased from 1.87 to 1.26 (F = 2.20; p = 0.14).

**Fig. 3:**
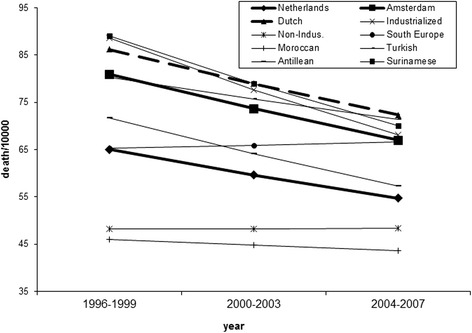
Chances of dying per 10,000 males aged between 45 and 65

Figure 3 shows the mortality per 10,000 males aged between 45 and 65. Again the trend is downward in both Amsterdam (z = 6.6; p < 0.01) and the Netherlands (z = 27.0; p < 0.01) but this time there is no difference in the trends of Amsterdam and the Netherlands (z = 0.5; p = 0.6). The ethnic groups mostly follow the improving trend. Again there is convergence between the groups, which is mainly caused by the already low mortality in groups from Morocco and the industrialized countries not changing much, while the mortality in most other groups decreased. No ethnic group changes statistically significantly different from the average for Amsterdam. The standard deviation decreased from 17.4 to 11.1 between 1996–1999 to 2004–2007 (F = 2.46; p = 0.11).

**Fig. 4:**
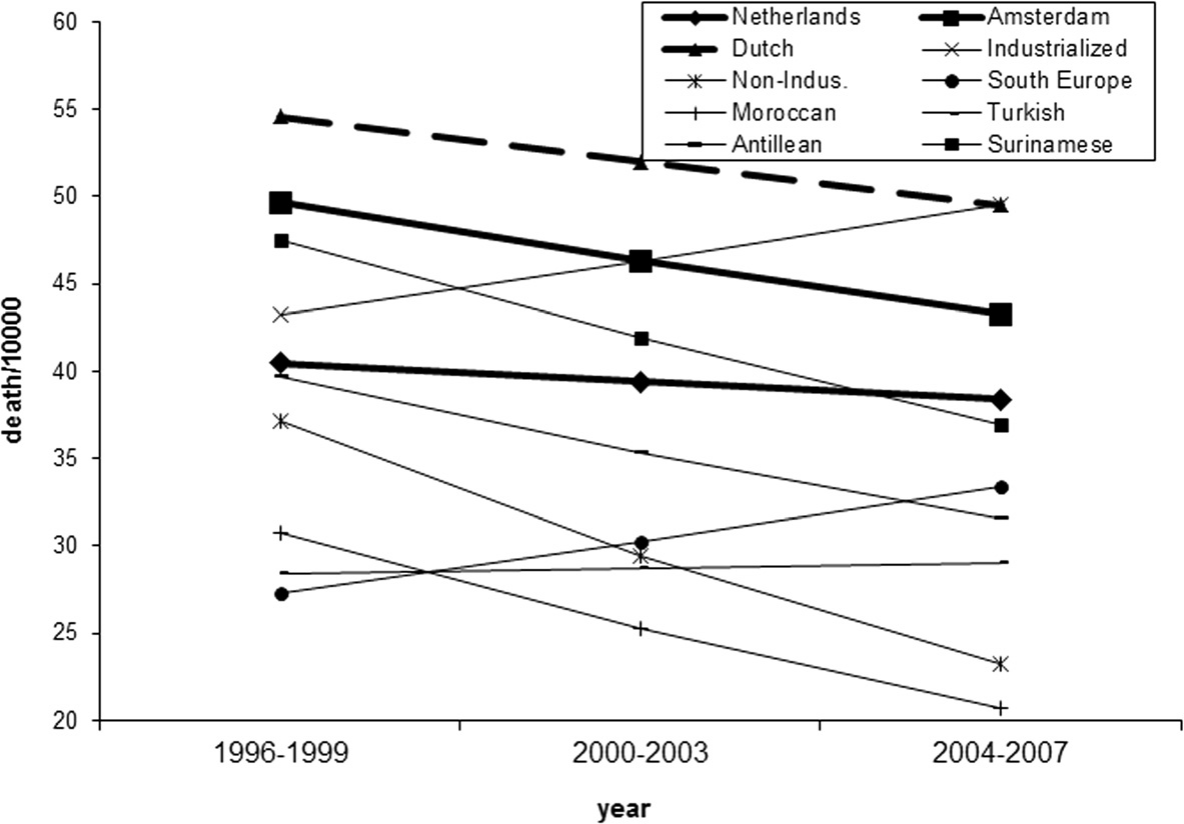
Chances of dying per 10,000 females aged between 45 and 65

Figure 4 shows the trend lines for mortality in females aged between 45 and 65 years. Again, mortality goes down in both Amsterdam (z = 6.7; p < 0.01) and the Netherlands (z = 3.7; p < 0.01), and more so in Amsterdam (z = 2.2; p < 0.03). The downward trend is followed by most groups, going down in Dutch, Surinam, Antillean females and females from non-industrialized and industrialized countries, and up in the other three ethnicities. Compared with the average trend for Amsterdam only the trend for females from the industrialized countries is significant (z = 2.4; p < 0.02). There was no difference between the Poisson and the negative binomial model (Chi2 < 0.01; p = 0.96). Note that the mortality in females aged between 45 and 65 years in Amsterdam as a whole is strongly influenced by the (high) mortality in females of Dutch ethnicity (bold dashed line). Lastly, there is a small divergence between the migrant groups in the mortality in females aged between 45 and 65, the standard deviation increasing from 9.7 to 10.8 (F = 1.24; p = 0.38).

Table 3 shows the life expectancy at age 65. For this oldest age group the life expectancy is chosen instead of the probability of death because all people inevitably die above the age of 65 giving a probability of death of a 100 %. The life expectancy increased both in the Netherlands and Amsterdam, in the bottom two rows, for both males and females. The rate of increase seems stronger for males than for females. The increase is followed by most groups, with the exception of males from Surinam, the Antilles and Southern Europe, and females from Turkey, Morocco and Southern Europe. It is notable that female citizens of Dutch descent, by far the largest group, have the lowest life expectancy at 65 of all females, while Dutch males have one of the lowest life expectancy. The standard deviation for the life expectancies for males increased from 2.2 1996–1999 to 3.5 in 2004–2007 (F = 2.53; p = 0.11), whereas for females it decreased from 10.0 to 1.7 (F = 34.6; p < 0.01).

**Table 3:** Life expectancy at age 65, Amsterdam 1996–2007 (95 % confidence interval)

	*Males*			*Females*		
	2004-2007	2000-2003	1996-1999	2004-2007	2000-2003	1996-1999
Dutch	16.1 (15.9-16.3)	15.1 (14.9-15.3)	14.7 (14.5-14.9)	19.5 (19.3-19.6)	18.5 (18.4-18.7)	18.6 (18.5-18.8)
Industrialized	16.9 (16.4-17.3)	15.3 (14.8-15.8)	14.9 (14.4-15.4)	19.7 (19.3-20.1)	18.1 (17.7-18.5)	18.6 (18.2-19.0)
Non Industrialized	19.1 (18.1-20.1)	17.0 (16.0-18.1)	16.4 (15.2-17.6)	23.0 (22.0-24.1)	20.4 (19.6-21.3)	20.0 (19.0-21.0)
Southern European	17.3 (16.3-18.3)	17.4 (16.1-18.7)	18.3 (16.5-20.2)	23.1 (21.5-24.7)	22.5 (21.2-23.7)	24.7 (22.4-26.9)
Moroccan	26.3 (24.7-27.9)	19.3 (17.9-20.7)	20.0 (18.2-21.8)	23.8 (22.4-25.3)	23.2 (20.5-25.9)	42.2 (36.9-47.6)
Turkish	15.9 (14.8-17.1)	16.6 (14.2-18.9)	13.6 (11.5-15.7)	20.4 (19.1-21.7)	20.9 (18.9-22.8)	40.9 (35.5-46.3)
Antillean	15.9 (13.8-18.1)	16.7 (13.4-20.0)	18.8 (15.1-22.5)	23.1 (21.0-25.1)	23.0 (20.1-25.9)	20.3 (17.9-22.8)
Surinamese	17.1 (16.3-17.8)	17.2 (16.3-18.0)	17.2 (16.2-18.1)	21.1 (20.4-21.7)	19.6 (18.9-20.3)	20.3 (19.5-21.1)
Standard dev.	3.49	1.31	2.24	1.73	1.99	9.97
City of Amsterdam	16.4 (16.2-16.5)	15.2 (15.1-15.4)	14.8 (14.7-15.0)	19.6 (19.5-19.8)	18.6 (18.4-18.7)	18.7 (18.6-18.8)
Netherlands	16.8 (16.8-16.9)	15.7 (15.6-15.7)	15.0 (15.0-15.1)	20.6 (20.6-20.6)	19.5 (19.5-19.6)	19.4 (19.4-19.4)

## Discussion

In this paper standard mortality data is used to study differences in mortality between eight ethnic groups living in Amsterdam, the Netherlands. The emphasis in this study is placed on changes in mortality between these groups during three periods, 1997–2000, 2001–2003 and 2004–2007. Particularly at interest is the question to what extent differences between groups converge as a possible indicator of health acculturation. A major limitation of this study is that mortality, particularly among more recent immigrant groups, is relatively low. In various ways this might influence mortality and life table calculations. One problem is that there will be considerable chance fluctuation in the data which will increase variance, lower the precision and reliability of any comparison made, which in the case of this study might lead to a low estimate in the F-test in comparing the standard deviations. Another problem is that the population might not be equally spaced in the age categories. For life expectancy calculations this is a particular problem in the highest age category, 85+ in the case of this study. If a population 85+ is relatively young, than there will be few deaths relative to the size of the population 85+, leading to a high calculated life expectancy overall. The instability in the life expectancy might increase the value of the standard deviation in comparing time periods. Lastly, the time series discussed in this paper is rather short and does not allow for long term conclusions to be drawn.

However, the outcomes of this study are in accordance with similar studies done on larger datasets and for longer periods and allows for some interesting conclusions to be drawn. In this study mortality trends - in terms of decreasing standard deviations – tend to converge between 1997–2000 and 2004–2007. The exceptions are life expectancy for males, particularly males aged above 65 years, and mortality risks among females between 45 and 65 years, where standard deviations increase, but not statistically significantly. This is also found for the Netherlands in general [16]. As in the earlier Dutch study, the data presented here shows the remarkable tendency that trends in mortality and life expectancy apply to all groups. Autonomous mortality trends in migrant groups seem almost negligible compared with the influence of much more powerful common trends. This observation has been the topic of some speculation, as it seems rather unlikely that, given the large cultural differences between the groups, developments in lifestyle in each cultural group independently can explain these common trends. Improvements in health care are suggested as an explanation [16]. In addition to the influence of health care it is possible that societal change is another important factor. For example, trauma mortality is an important factor in the mortality in younger age groups, particularly in ethnic minorities [20, 21]. In this context all groups in society benefit from safer environments, safer tools and equipment, lower speeds and more policing. Similarly, increased societal affluence might make it easier for all groups to behave more healthily; however, each group might change in its own culturally distinctive way. One wonders if bridging the mortality gap between groups is of much benefit for minority groups, or that groups would benefit more from an overall decrease in mortality.

As in previous studies, the remarkable “healthy migrant effect” can be clearly observed. The longer established Dutch population, who generally live in better houses, who have better jobs and more opportunities, show, contrary to expectation, a lower life expectancy. Among women between the ages 45 to 65 women of Dutch origin have the highest mortality expectation of the ethnic groups and they also have the lowest life expectancy from age 65 onwards. Compared with females, males of Dutch origin fare slightly better. The effect of the local population performing worse compared with the more recently established migrant population, is found worldwide and has been the topic of much debate [4, 22–28]. It is important to realize that in a city like Amsterdam the healthy migrant effect operates differently compared with a nation state. Here migration plays a dual role. Simultaneous with the immigration of peoples from abroad, there is a large emigration of the original Dutch population to suburban areas [25]. The total size of the population of the city has hardly grown [27]. The ethnic Dutch emigrants are often in young families, relatively affluent, generally more health-conscious and with a better general health [28]. This has a profound effect on the population in Amsterdam of Dutch origin, depriving this population of economically important groups and many talented youth. The result is that both males and females of Dutch origin living in Amsterdam have a considerably lower life expectancy compared with the Netherlands at large. As most of the population of the Netherlands is made up of people of Dutch descent also, this difference is caused not by ethnicity but by selection. This process of selection is continuing but now in the migrant population. As sections of longer established immigrants become more affluent and better educated, they too migrate to the suburbs, leaving those in a lesser social position and more recent immigrants behind [17, 29].

## Conclusion

This paper is limited to mortality and does not allow for strong conclusions concerning other aspects of health, nor does it provide a deep insight in the causes of changes in mortality over time. What the paper does show is that there is a certain level of convergence between various migrant groups in terms of mortality, however, that the improvement in mortality and life expectancy involving all groups is the more powerful force. Further study could look at trends in mortality in various immigrant groups by cause of death. It would be interesting to see if among migrants trauma becomes less common, or cancers more common, with an increasing length of stay in the Netherlands.

## References

[1] Berry JW (1997). Immigration, Acculturation, and Adaptation Applied. Psychology.

[2] Berry JW, Chun KM, Organista PB, Marín G (2003). Conceptual approaches to acculturation. Acculturation: Advances in theory, measurement and applied research.

[3] Phinney JS, Horenczyk G, Liebkind K, Vedder P (2002). Ethnic Identity, Immigration, and Well-Being: An Interactional Perspective. J Soc Issues.

[4] Uitenbroek DG, Verhoeff AP (2002). Life expectancy and mortality differences between migrant groups living in Amsterdam. The Netherlands Soc Sci Med.

[5] Hunt LM, Schneider S, Comer B (2004). Should "acculturation" be a variable in health research? A critical review of research on US Hispanics. Soc Sci Med.

[6] Landrine H, Klonoff E (2004). Culture change and ethnic-minority health behavior: an operant theory of acculturation. J Behav Med.

[7] Bos V, Kunst AE, Garssen J, Mackenbach JP (2007). Duration of residence was not consistently related to immigrant mortality. J Clin Epidemiol.

[8] Graves TD (1967). Psychological acculturation in a tri-ethnic community. Southw J Anthropol.

[9] Hammar N, Kaprio Hagström U, Alfredsson L, Koskenvuo M, Hammar T (2002). Migration and mortality:a 20 year follow up of Finnish twin pairs with migrant co-twins in Sweden. J Epidemiol Community Hlth.

[10] Bos V, Kunst AE, Keij-Deerenberg IM, Garssen J, Mackenbach JP (2004). Ethnic inequalities in age- and cause-specific mortality in the Netherlands. Int J Epidemiol.

[11] Parker Frisbie W, Cho Y, Hummer RA (2001). Immigration and the Health of Asian and Pacific Islander Adults in the United States. Am J Epidemiol.

[12] Singh GK, Hiatt RA (2006). Trends and disparities in socioeconomic and behavioural characteristics, life expectancy, and cause-specific mortality of native-born and foreign-born. Int J Epidemiol.

[13] Meara ER, Richards S, Cutler DM (2008). The gap gets bigger: Changes in mortality and life expectancy, by education 1981–2000. Hlth Affairs.

[14] Firebaugh G, Acciai F, Noah AJ, Prather CJ, Nau C (2014). Why the racial gap in life expectancy is declining in the United States. Demogr Res.

[15] Harper S, MacLehose RF, Kaufman JS (2014). Trends in the black-white life expectancy gap among US states, 1990–2009. Hlth Aff (Millwood).

[16] Garssen J, van der Meulen A (2007). Overlijdensrisico’s naar herkomstgroep: daling en afnemende verschillen. CBS Bevolkingstrends.

[17] Zorlu A, Hartog J, Rotte R, Stein P (2002). Migration and immigrants: The case of the Netherlands. Migration Policy and the Economy: International Experiences.

[18] Alders M (2001). Classification of the population with a foreign background in the Netherlands.

[19] Chiang CL, Chiang CL (1968). The life table and its construction. Introduction to Stochastic Processes in Biostatistics.

[20] Uitenbroek DG, Van der Wal M, Van Weert-Waltman L (2000). The effect of a health promotion campaign on mortality in children. Hlth Educ Res.

[21] Stirbu I, Kunst AE, Bos V, Mackenbach JP (2006). Injury mortality among ethnic minority groups in the Netherlands. J Epidemiol Community Hlth.

[22] Khlat M, Darmon N (2003). Is there a Mediterranean migrants mortality paradox in Europe?. Int J Epidemiol.

[23] Razum O (2006). Commentary: Of salmon and time travellers--musing on the mystery of migrant mortality. Int J Epidemiol.

[24] Palloni A, Arias E (2004). Paradox lost; explaining the Hispanic adult mortality. Demography.

[25] van Huis M, Nicolaas H, Croes M: Migration of the four largest cities in the Netherlands. Voorburg: CBS, undated.

[26] Wallace M, Kulu H (2014). Low immigrant mortality in England and Wales: a data artefact?. Soc Sci Med.

[27] Ikram UZ, Mackenbach JP, Harding S, Rey G, Bhopal RS, Regidor E, Rosato M, Juel K, Stronks K, Kunst AE: All-cause and cause-specific mortality of different migrant populations in Europe. Eur J Epidemiol 2015, [Epub ahead of print] 10.1007/s10654-015-0083-9 PMC4977342 26362812

[28] O&S: Demografische ontwikkelingen. O&S Factsheet 2004, 6. https://www.ois.amsterdam.nl/pdf/2004_factsheets_6.pdf

[29] Verheij RA, van de Mheen HD, de Bakker DH, Groenewegen PP, Mackenbach JP (1998). Urban–rural variations in health in The Netherlands: Does selective migration play a part?. J Epidemiol Community Hlth.

